# The developmental high wire: Balancing resource investment in immunity and reproduction

**DOI:** 10.1002/ece3.8774

**Published:** 2022-04-06

**Authors:** Daniel J. Breiner, Matthew R. Whalen, Amy M. Worthington

**Affiliations:** ^1^ 6216 Department of Biological Sciences Creighton University Omaha Nebraska USA; ^2^ 1259 Department of Psychology University of Michigan Ann Arbor Michigan USA

**Keywords:** cricket, development, fecundity, fitness, *Gryllus*, immunity, life history trade‐offs, reproduction

## Abstract

The strategic allocation of resources into immunity poses a unique challenge for individuals, where infection at different stages of development may result in unique trade‐offs with concurrent physiological processes or future fitness‐enhancing traits. Here, we experimentally induced an immune challenge in female *Gryllus firmus* crickets to test whether illness at discrete life stages differentially impacts fitness. We injected heat‐killed *Serratia marcescens* bacteria into antepenultimate juveniles, penultimate juveniles, sexually immature adults, and sexually mature adults, and then measured body growth, instar duration, mating rate, viability of stored sperm, egg production, oviposition rate, and egg viability. Immune activation significantly impacted reproductive traits, where females that were immune challenged as adults had decreased mating success and decreased egg viability compared to healthy individuals or females that were immune challenged as juveniles. Although there was no effect of an immune challenge on the other traits measured, the stress of handling resulted in reduced mass gain and smaller adult body size in females from the juvenile treatments, and females in the adult treatments suffered from reduced viability of sperm stored within their spermatheca. In summary, we found that an immune challenge does have negative impacts on reproduction, but also that even minor acute stressors can have significant impacts on fitness‐enhancing traits. These findings highlight that the factors affecting fitness can be complex and at times unpredictable, and that the consequences of illness are specific to when during an individual's life an immune challenge is induced.

## Graphical abstract

The strategic allocation of resources into immunity poses a unique challenge for individuals, where infection at different stages of development may result in unique trade‐offs with concurrent physiological processes or future fitness‐enhancing traits. We experimentally induced an immune challenge in female *Gryllus firmus* crickets to test whether illness at discrete life stages differentially impacts fitness, including such traits as body growth, instar duration, mating rate, viability of stored sperm, egg production, oviposition rate, and egg viability.

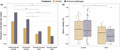

## 1 INTRODUCTION

Resource competition among life history traits is multi‐dimensional and ubiquitous across animal taxa. Life history trade‐offs operate under the principle that investing in one trait comes at a detriment to investment in other traits, especially when energetic resources are limited (Stearns, 1989). Although such trade‐offs are usually described by the Y allocation model (Van Noordwijk & de Jong, 1986), trade‐offs are rarely confined between just two processes. Trait investment then becomes an issue of efficiency and optimization in order for an organism to maximize their fitness given the available resources and the specific conditions encountered within the environment (Stearns, 2000). Apart from self‐maintenance, three energetically costly processes organisms rely on for obtaining fitness are somatic growth (Roff, 1992), reproduction (Edward & Chapman, 2011), and immunity (McKean & Lazzaro, 2011). Investments into these traits are paramount for an individual's fitness and must be done prudently given that trade‐offs between somatic growth and reproduction (Rohwer et al., 2011), reproduction and immunity (Krams et al., 2017), and immunity and somatic growth (Jacot et al., 2005a) are observed across the animal kingdom.

In particular, the strategic allocation of resources into immunity poses a unique challenge for individuals. Neglecting to invest in immunity comes at the risk of premature death should infection occur; however, sustaining a high level of immunocompetence during healthy periods requires energy (Schmid‐Hempel, 2005) that may be better directed to other fitness‐enhancing traits. Furthermore, prudently investing in immunity is difficult due to the unpredictable nature of when a life‐threatening infection could occur during an individual's life. Therefore, individuals at different stages of development may experience unique trade‐offs upon upregulating their immune system depending on what other processes are concurrent at that time. For example, during periods of growth, infection may cause resources to be diverted to immunity rather than the growth of bodily structures, as seen in juvenile *Drosophila melanogaster* infected with parasitic wasp larvae (Fellowes et al., 1999). Alternatively, infections occurring after reproductive maturity has been reached are more likely to influence reproductive physiology (Radhakrishnan & Fedorka, 2012) or courtship behaviors (Polak & Starmer, 1998) since somatic growth is complete.

Individuals mediate the negative impacts of resource competition among their traits in varied ways. Following a physiologically costly event or a period of poor nutrition, juveniles may increase their growth rate (i.e., compensatory growth) or prolong development (i.e., catch‐up growth) to increase body size prior to maturity (Jobling, 2010; Metcalfe & Monaghan, 2001). For example, female field crickets deprived of food will prolong development time in order to maximize their adult body size (Tawes & Kelly, 2017), an important determinant of lifetime fitness (Saleh et al., 2014). Should infection occur after sexual maturity, however, considerable strain is placed on gamete production upon upregulating the immune response (Schwenke et al., 2016). Rather than invest in disease resistance, individuals may forgo immune upregulation to instead maximally invest in reproductive efforts in a process called terminal investment (Adamo, 1999; McNamara & Houston, 1996). In adult burying beetles (*Nicrophorus vespilloides*), females will increase reproductive investment and produce heavier broods when given an immune challenge perceived to be threatening to their survivorship compared to controls (Cotter et al., 2011). The timing of an infection therefore has a critical impact on the strategic allocation of resources, thus determining which trade‐offs with key life history traits an individual will experience.

Field crickets (*Gryllus* sp.) are surprisingly well‐studied regarding life history trade‐offs, and our general knowledge of their development, physiology, and reproduction provides a strong foundation for investigating questions related to the time‐specific costs of infection. *Gryllus* crickets demonstrate a robust immune response to the many parasites, pathogens, and other immune challenges they may encounter during development (Jacot et al., 2005b; Kirschman et al., 2019). Additionally, they are hemimetabolous and extensive growth occurs in the last two juvenile instars, a time when wing buds finally become visible and body size increases dramatically (unpublished data from our lab indicates that on average 65% of total adult mass (min–max range = 57–71%) is gained in the last two instars). Interestingly, *Gryllus* exhibits flexible patterns in development where it may take individuals 8–12 instars to go from egg to adult (Jobin, 1961), such that crickets that encountered illness or energy restrictions as juveniles can undergo compensatory or catch‐up growth by prolonging development so that final adult size remains unaffected (Tawes & Kelly, 2017). In addition to somatic growth, juvenile female field crickets must accumulate large energy‐rich fat stores to fuel ovary growth and egg production in the first week of adulthood (Lorenz, 2007), after which they become sexually receptive to mating (Worthington & Kelly, 2016a). Females mate repeatedly as adults to maintain viable sperm within their spermatheca with which to fertilize their numerous eggs (Worthington & Kelly, 2016a), and bacterial infection by *Serratia marcescens*, especially in nutrient‐restricted females, negatively impacts the viability of stored sperm within 2 days of infection (McNamara et al., 2014). This is likely due to condition‐dependent trade‐offs between clearing an infection and the ability to maintain high sperm viability within the spermatheca, which was also directly impacted by resource limitation even in the absence of an immune challenge. Thus, the impacts of infection on reproduction in female crickets is confounding and likely situation specific, depending on additional factors such as age, nutrition, infection intensity, infection duration, and even environmental temperature. For example, females infected with a controlled dose of a pathogen have been shown to increase reproductive investment – indicative of terminal investment (Shoemaker et al., 2006a), maintain reproductive investment (Adamo & Lovett, 2011; Shoemaker & Adamo, 2007; Shoemaker et al., 2006a), or reduce reproductive investment – demonstrating a resource trade‐off with immunity (Adamo & Lovett, 2011; Stahlschmidt et al., 2013). Although these individual life history trade‐offs with illness are well studied, we still have little understanding of when during development infection has the largest impact on overall fitness, in part because past studies have generally focused on trade‐offs occurring at just one infection time point.

Here, we use the sand field cricket, *Gryllus firmus*, to experimentally test whether illness at discrete developmental stages has unique trade‐offs with concurrent and/or future physiological processes, and we directly compare the overall fitness of individuals that experienced immune challenges at different points in their development. To do this, we exposed female crickets to a non‐lethal bacterial challenge at one of four stages in development, and then quantified changes to their subsequent growth and reproductive output. We hypothesized that the traits most negatively affected for each time treatment would be those that individuals are investing in concurrent to the upregulation of their immune response. Specifically, we predicted that immune‐challenged juveniles would have decreased body growth or experience longer development times, and alternatively that immune‐challenged adults would experience trade‐offs directly related to reproduction, such as egg production, viability of stored sperm, oviposition, or egg viability. If evidence of age‐dependent life history trade‐offs exist, our results will allow us to identify which developmental stages are most sensitive to reductions in fitness due to infection.

## 2 METHODS

### 2.1 Animal housing

We used *Gryllus firmus* crickets taken from an artificially selected population of nearly pure‐breeding short‐wing (SW) adults. Crickets in this SW‐selected population immediately and more heavily invest in reproduction upon eclosion into flightless adults (Roff, 1984; Zera & Denno, 1997) and are characterized by having differential investment in immune response relative to the long‐winged (LW) flight‐capable morph (Kirschman et al., 2019). We exclusively used SW individuals because the differences in life histories between the two wing morphs would confound the results of reproductive and immune investment across treatments. Additionally, LW‐destined crickets regularly eclose into SW adults under challenging environmental conditions such as those used in the study (Shizmu & Maskai, 1993), thus we preemptively controlled for this unpredictable factor using only SW‐selected individuals.

Laboratory populations were reared in 85‐L clear plastic bins with ventilated lids. Here, crickets were supplied stacks of egg cartons for structure, fed Special Kitty Premium cat food ad libitum, and provided large cotton‐plugged water vials. Experimental female crickets were sorted individually into 350‐ml deli cups early in their antepenultimate instar with 2–3 weeks remaining of juvenile development. Here, they were provisioned with a small cardboard shelter, a small cotton‐plugged water vial, and dry cat food ad libitum. All crickets were housed in an environmentally controlled room (26–28°C; 70–80% humidity; and 12:12 h light/dark cycle).

### 2.2 Experimental design

We randomly divided antepenultimate females into either the control or immune‐challenged treatment group, and each female was assigned one of four time points to receive their treatment: (1) antepenultimate, (2) penultimate, (3) sexually immature, or (4) sexually mature. Antepenultimate females received their treatment on the first day of the experiment when they were two molts away from adulthood, as indicated by small wing buds and an ovipositor extending only 1–2 mm past the end of the abdomen. Penultimate females received their treatment 5 days after molting into their final juvenile instar, and were identified by their large wing buds and an ovipositor approximately 5–6 mm in length. Sexually immature females received treatment 2 days after eclosing into adults once their exoskeleton had fully hardened to prevent excess injury while receiving their treatment. At this early adult stage, a females’ eggs are only just beginning to develop within the ovaries and females have low receptivity to mating (Solymar & Cade, 1990). Finally, sexually mature females received treatment 7 days after eclosing into adults – a time when ovaries are full of developed eggs and females become receptive to mating (Worthington & Kelly, 2016a).

At their designated treatment time, crickets were cold anesthetized for 3:15, 3:30, or 4:00 min for antepenultimates, penultimates, and adults, respectively. To elicit a non‐lethal yet robust immune response, we sterilized the abdomens of immune‐challenged crickets with 70% ethanol and inserted a sterile glass microcapillary needle between the second and third abdominal sclerites to inject 1.0 × 10^4^ cells/5 μl of the heat‐killed bacterium *Serratia marcescens* (obtained as a live Microkwik culture from Carolina Biological Supply #155450A and diluted to concentration with phosphate‐buffered saline). This number of cells is equivalent to an LD_50_ dose of live *S*. *marcescens* (Worthington & Kelly, 2016b), however, we used heat‐killed *S*. *marcescens* to avoid the pathogenic effects of live bacteria while still effectively activating the immune response (Adamo, 2004; Stahlschmidt et al., 2015) and inducing sickness behavior (Adamo et al., 2010). We plated heat‐killed *S*. *marcescens* to test for viability, and no live colonies were ever observed after exposure to heat. Control females were anesthetized, sterilized, and handled underneath the stereoscope, but did not receive any injection. After receiving their treatment, females were returned to their original containers and monitored until they recovered.

All females were reared individually until they reached their ninth day of adulthood, at which time each female was placed into a 1.2‐L container with a randomly assigned healthy adult male to mate with and provisioned with a cardboard shelter, a cotton‐plugged water vial, and a piece of cat food. Mated adult males 2–3 weeks post‐eclosion were randomly chosen from our breeding population. Only males of average size were paired with females, as obviously small and large males were avoided during selection. Each male was used only once. After 24 h, the male was removed and a small cup of moistened fine sand (Reptilite, Ft. Pierce, FL, USA) was added for the female to oviposit into. Females were given 48 h to oviposit before the trial was ended on the 12th day of adulthood and females were processed for the presence and viability of sperm stored in their spermatheca, as well as the number of eggs present in their ovaries.

### 2.3 Growth & development

Mass (to the nearest 0.01 mg) and pronotum length (the distance between the anterior and posterior edges at the midline) were measured for the antepenultimate crickets on the first day of the experiment, and then again on the first day of the penultimate and adult instars. Each cricket was photographed at 0.75× magnification using a Leica IC90‐E camera mounted on a Leica M80 stereoscope, then pronotum was digitally measured to the nearest 0.001 mm using LAS Core Software (Version 4.9). Crickets were monitored daily for molting or death, and the number of days that each individual spent in the penultimate instar was calculated from the dates we recorded. Food and water were replaced only as needed to minimize disturbance.

### 2.4 Sperm viability within spermatheca

On day 12 of adulthood, females were cold anesthetized for 5 min so we could dissect their spermatheca to perform a sperm viability assay. The LIVE/DEAD^TM^ assay (Molecular Probes, Eugene, OR, USA) stains live sperm green using SYBR^®^‐14 and stains dead sperm red using propidium iodide, and has been effectively used to quantify viability on sperm recovered from spermatheca (McNamara et al., 2014). After dissection, we placed each spermatheca in 20 µL of Beadle's saline (128.3 mM NaCl, 4.7 mM KCl, and 23 mM CaCl_2_) and gently ruptured with fine forceps. Following 10 min of incubation, we added 5 µl of 1:50 SYBR^®^‐14 solution (1.25 µl SYBR^®^‐14 in 50 µl Beadle's Saline), then incubated the solution in the dark for 5 min before adding 2.5 µl of propidium iodide and incubating in the dark for an additional 5 min. Following this final incubation, we pipetted 10 µl of the solution into each well of a disposable hemocytometer (INCYTO C‐Chip, Covington, GA, USA). Sperm were visualized at 400× magnification on a fluorescent microscope (Leica DM2000 LED, Leica Microsystems GMBH, Wetzlar, Germany). Sperm located within five predetermined squares of the grid were counted as living (fluoresced green) or dead (fluoresced red). All sperm counts were made by D.J.B., who was blind to experimental treatment at time of assay. Sperm viabilities are reported as the percentage of viable sperm within the spermatheca (i.e., the number of live sperm divided by the total number of sperm).

### 2.5 Fecundity & egg viability

At the same time that the spermatheca was dissected, the total number of eggs contained within the ovaries was quantified. To approximate maternal investment egg size, five fully developed eggs (i.e., those most posterior) from the right ovary were imaged and their length recorded. Upon dissecting the female on day 12, the oviposition egg cup was maintained at 27°C for a further 9 days to allow the oviposited eggs to develop. The moist sand was then air dried for 24 h so the eggs could be collected using a fine mesh sieve. We quantified both the total number of eggs laid and the proportion of those eggs that were viable. Eggs were only considered viable if they had eye spots after 10 days of development. Fecundity was calculated as a sum of the eggs found within the lateral oviducts and the total number of eggs that were oviposited.

### 2.6 Statistical analyses

All analyses were conducted in R (v3.6.3; R Core Team, 2013). All traits were analyzed using generalized linear models (GLM) or generalized linear mixed models (GLMM) using the package lme4 (Bates et al., 2007). Continuous covariates including mass and pronotum length were scaled to a mean of 0 and standard deviation of 1. This was done separately for each model due to sample size differences for different measures of fecundity. For each model, we included time of treatment, treatment, and their interaction as fixed effects. For binomial and Poisson models, an observation‐level random effect was included to account for overdispersion in the data (Harrison, 2014). Global means for all models were assessed using the ANOVA function in the car package (Fox, 2006). When a significant main effect was found, differences among treatment groups in each model were compared by estimating their marginal means through the emmeans package (Lenth & Lenth, 2018) with a Tukey adjustment for multiple comparisons.

For measures of sperm viability and egg viability, missing values in the dataset due to unmated females gave unreliable coefficient estimates. Instead, we grouped individuals that were treated during their antepenultimate and penultimate instars together as the “juvenile” group and we grouped sexually immature and sexually mature adults together as the “adult” group. As a result, we report here differences between treatment (control or immune challenged) and development stage (juvenile or adult) and their interaction (Treatment x Developmental Stage) for these traits.

## 3 RESULTS

### 3.1 Growth & development

**FIGURE 1:**
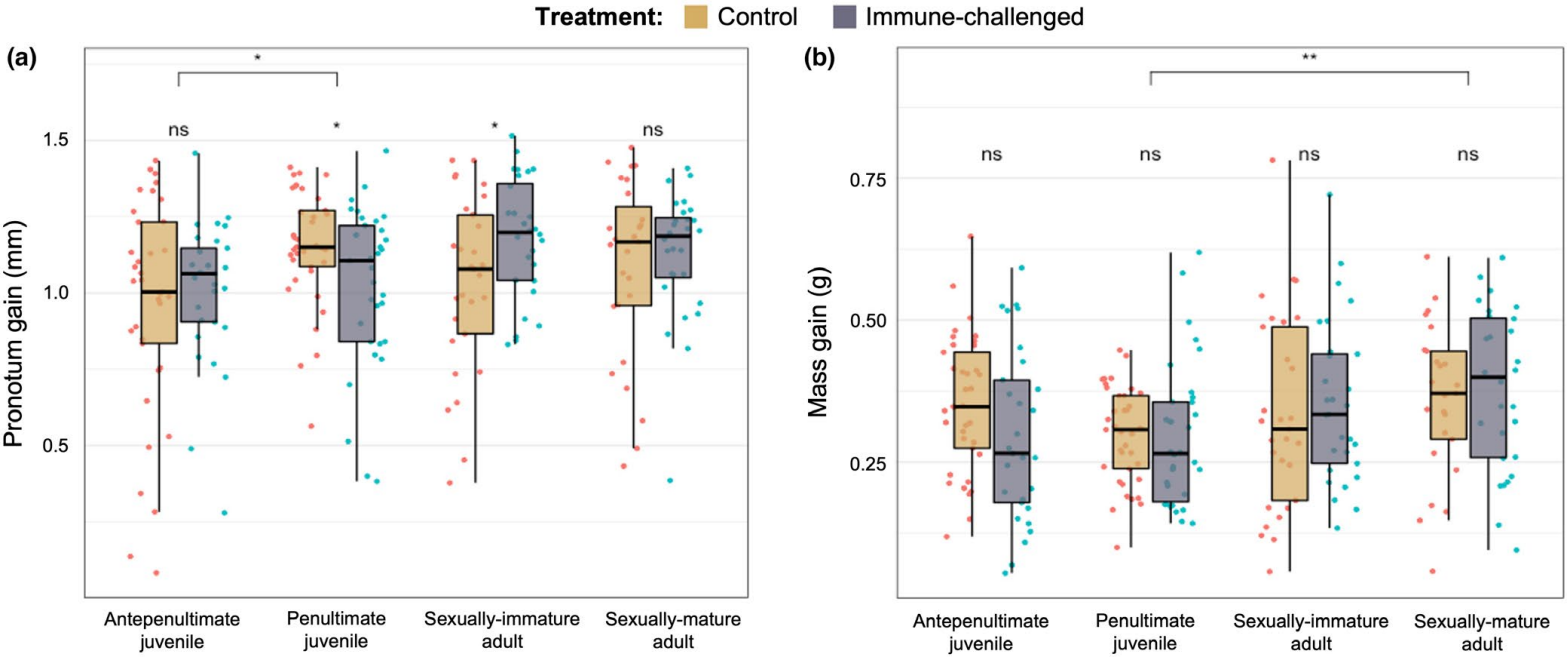
Pairwise comparisons for body measurements across all groups that received their treatment at the developmental stages listed: (a) mass gained during penultimate instar, and (b) pronotum length gain during penultimate instar. Control individuals are shown in yellow, while immune‐challenged individuals are shown in purple

There was no effect of Treatment (*F*_1,244_ = 0.0163, *p* = .899), Time (*F*_3,244_ = 1.844, *p* = .140), or their interaction (*F*_3,244_ = 1.655, *p* = .177) on the duration of penultimate instar (*N* = 244). There was a significant effect of Time on mass gained (*N* = 248) from the antepenultimate instar to the first day of adulthood (*F*_3,240_ = 3.367, *p* < .05; Table 1), however, there was no effect of Treatment (*F*_1,240_ = 0.177, *p* = .675). Pairwise comparisons showed that both control and experimental females handled at the beginning of their penultimate instar showed significantly less mass gain than sexually immature females handled on the second day of adulthood (Figure 1a). For pronotum growth (*N* = 244), we found a significant effect of Time (*F*_3,235_ = 3.177, *p* < .05) as well as a significant interaction between Treatment and Time (*F*_3,240_ = 3.735, *p* < .05; Table 1). Pairwise comparisons revealed that across treatments, both control and experimental females treated as antepenultimates showed significantly less pronotum gain than those treated as penultimates (difference ± *SE* = 0.109 ± 0.0378; Figure 1b). Within the control treatment, females handled as antepenultimates (difference ± *SE* = 0.198 ± 0.515) and sexually immature adults (difference ± *SE* = 0.1525 ± 0.540) showed significantly less pronotum growth than females handled as penultimates. There were no significant pairwise differences within the experimental group.

**TABLE 1:** Summarized unreduced models for individuals treated across all four developmental stages

Fixed effect	Mating success		Total eggs		Eggs laid		Mass gained		Pronotum gain	
	χ^2^	*p*	χ^2^	*p*	χ^2^	*p*	*F* value	*p*	*F* value	*p*
Time	32.4	<.001	8.01	<.05	1.21	.750	3.40	<.5	3.21	<.05
Treatment	4.41	<.05	1.58	.209	0.17	.680	0.176	.67	0.180	.671
Time × Treatment	13.6	<.01	4.05	.256	0.72	.869	1.12	.34	3.73	<.05

### 3.2 Mating success & sperm viability

**FIGURE 2:**
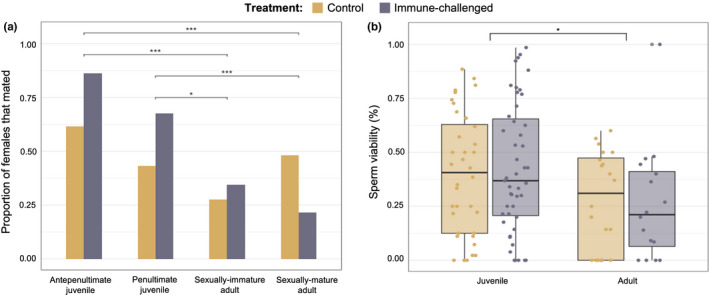
Mating success and sperm viability by developmental stage at which treatment was administered: (a) pairwise comparisons of proportion of females in each treatment that successfully mated, and (b) proportion of viable sperm stored in spermatheca on day 12 of adulthood

There was a significant effect of Treatment (logistic regression: χ^2^ = 4.412, *df* = 1, *p* < .5), Time (χ^2^ = 32.407, *df* = 3, *p* < .001), and their interaction (χ^2^ = 13.60, *p* < .01) on the probability of a female mating at day 12 of adulthood (*N* = 244; Table 1; Figure 2a). Females immune challenged as juveniles showed higher mating success than females immune challenged as adults. Females immune challenged as antepenultimates had higher mating success than individuals immune challenged as sexually immature adults (difference ± SE = 2.811 ± 0.700, *p* < .001) and sexually mature adults (3.552 ± 0.745, *p* < .0001). Similarly, females immune challenged as penultimates had significantly higher mating success than females immune challenged at both the sexually immature (difference ± *SE* = 1.789 ± 0.576, *p* < .05) and sexually mature adult time points (2.530 ± 0.629, *p* < .001). There were no significant pairwise differences between females within the control treatment across time.

There was a significant effect of developmental stage (χ^2^ = 6.3645, *p* < .05), but not Treatment (χ^2^ = 0.242, *p* = .87), on the viability of sperm stored within the spermatheca (*N* = 113; Table 2; Figure 2b). Pairwise comparisons showed that control and immune‐challenged females treated as juveniles had significantly higher sperm viability than those treated as adults (estimate ± *SE* = 0.752 ± 0.298, *p* < .05).

**TABLE 2:** Summarized unreduced models for individuals treated as either juveniles or adults

Fixed effect	Sperm viability		Egg viability	
	χ^2^	*p*	χ^2^	*p*
Age	3.94	<.05	0.787	.375
Treatment	0.01	.910	0.192	.661
Age × Treatment	1.68	.195	4.05	.133

### 3.3 Egg production, size, number laid, and viability

**FIGURE 3:**
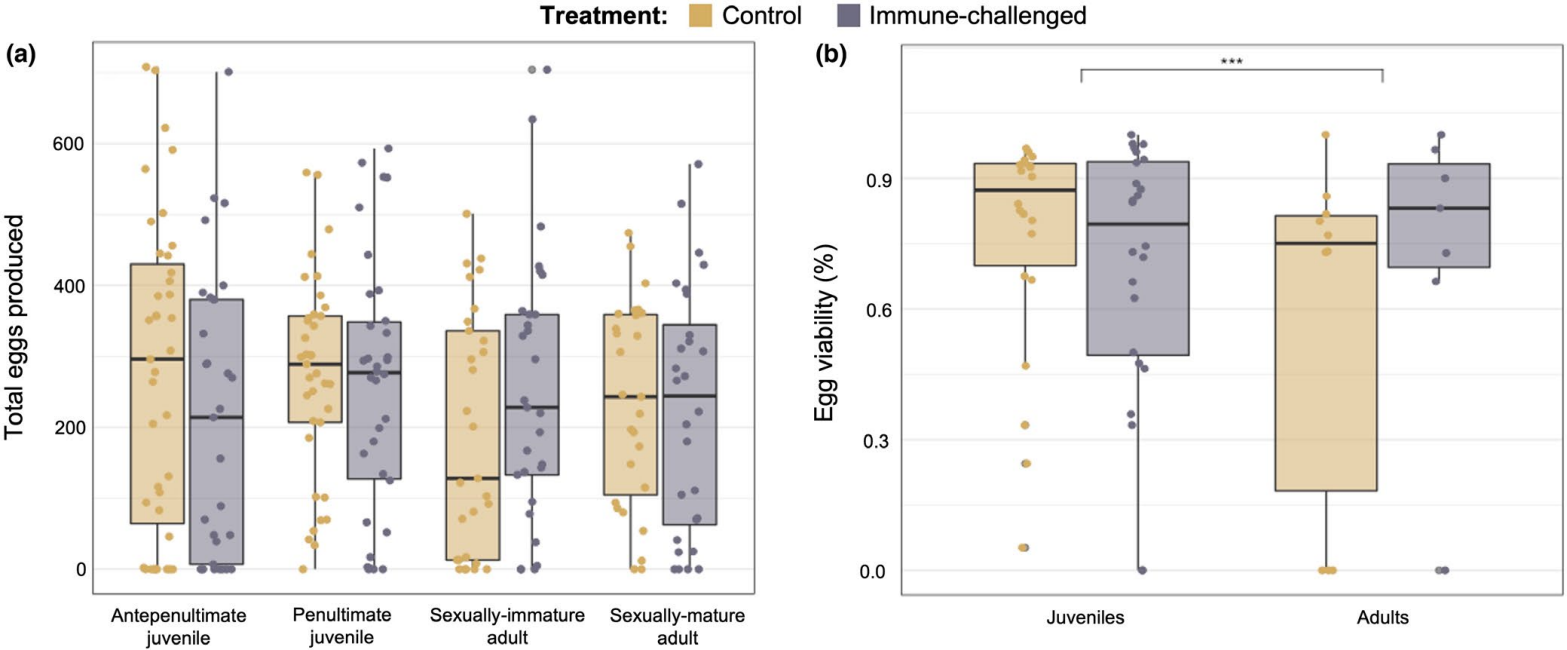
Fecundity and egg viability: (a) pairwise comparisons of total eggs contained produced by day 12 of adulthood for individuals that received their treatment at the developmental stages listed, and (b) proportion of viable eggs 10 days post‐oviposition for individuals that received their treatment as either juveniles or adults

We found no effect of Treatment (χ^2^ = 1.5576, df = 1, *p* = .2122) on the total number of eggs produced (*N* = 244; Table 1; Figure 3a). There was a weak effect of developmental treatment (χ^2^ = 7.922, *df* = 3, *p* < .05) on egg production, however, pairwise comparisons revealed no significant differences between groups. There was no effect of Treatment (χ^2^ = 0.170, *df* = 1, *p* = .680), Time (χ^2^ = 1.213 df = 3, *p* = .750), or their interaction (χ^2^ = 0.719, *df* = 1, *p* = .869) on the number of eggs laid per female (*N* = 215; Table 1). Prior to the analysis of mean egg length (*N* = 244), we identified and removed outliers. Removal of these outliers did not qualitatively change the results of the analysis. There was no effect of Treatment (*F*_3,195_ = 0.177, *p* = .674) or developmental stage on mean egg length (*F*_3,195_ = 0.379, *p* = .768).

Last, we found a significant Treatment effect (χ^2^ = 5.5704, *df* = 1, *p* < .05) on egg viability (Table 2; Figure 3b). Immune‐challenged females had significantly lower egg viability than control females (estimate ± *SE* = 0.594 ± 0.252, *N* = 61). Egg viability also differed significantly across developmental stage (χ^2^ = 21.9628, *df* = 1, *p* < .001), where females handled as adults had significantly lower egg viability than females treated as juveniles (estimate ± *SE* = 1.25 ± 0.267). There was no significant interaction between these Treatment and developmental stage (χ^2^ = 0.432, *df* = 1, *p* = .511).

## 4 DISCUSSION

We predicted that immune‐challenged female *G*. *firmus* would experience life history trade‐offs, specifically with physiological processes that were concurrent during the time of the immune challenge, and that illness could have long‐term negative impacts on future investments into reproduction. We found strong support that immune‐challenged adults indeed experience decreased mating success and decreased egg viability compared to healthy individuals or females that were immune challenged as juveniles. Surprisingly, however, a short‐term immune challenge had no effect on concurrent investment in juvenile body growth and development time, and neither concurrent nor future investment into stored sperm viability, egg size, egg production, and oviposition was affected in adults. Females therefore appear to be quite resilient to the negative fitness consequences of illness if their immune system is challenged as a juvenile, and adults experience fewer trade‐offs in reproductive traits than predicted by life history theory.

Our results concur with previous research that has widely failed to find evidence of terminal investment in response to non‐lethal immune challenges in female crickets (Miyashita et al., 2019); especially in scenarios when variation in resource acquisition is absent among individuals. However, Shoemaker et al. (2006a) did find that female *G*. *texensis* adults increase their oviposition rate in preferred substrates in response to a lethal bacterial infection, suggesting that females may be able to discriminate between lethal and sublethal immune challenges. This could be a result of the fact that sublethal immune challenges do not require as robust of an immune response and therefore have fewer physiological or resource trade‐offs, or because sublethal immune challenges are less likely to alter an individual's overall condition enough to trigger changes in resource allocation. Alternatively, females may not initiate terminal investment strategies until later in life when longevity is already waning or until changes to body condition are so drastic that death is imminent (Duffield et al., 2017). Alternatively, explicit tests of the Y allocation model in *Gryllus* have demonstrated that trade‐offs between energetically costly traits (e.g., ovarian mass and flight musculature) are most dramatic in situations when resource acquisition is limited, but may be completely absent when resources are plentiful (King et al., 2011). In our experiment, we provided food to females ad libitum, so they may have increased food intake to compensate for the increased costs of activating the immune response. However, a past study found that food‐limited females do not alter their reproductive investment when ill, even when nutrient intake is so low that it has a direct effect on the number of eggs produced and laid by non‐infected crickets (Miyashita et al., 2019).

That we saw no negative impact of an immune challenge on female ability to make and lay eggs is consistent with recent evidence that molecules important for immunity have other important physiological roles, resulting in unforeseen interactions between bodily systems (Adamo et al., 2008). For example, phenoloxidase (PO) has multifunctional roles in both the immune system and within the ovaries (Miyashita et al., 2019), and PO levels in female, but not male, crickets rise during adulthood when egg production begins (Adamo et al., 2001). This increase in PO levels could reduce competition for this physiologically important molecule and prevent large trade‐offs between egg production and immunocompetence at a time critical to maximizing female fitness. Additionally, female crickets acquire fitness‐enhancing compounds from the spermatophores of males while mating, and these have been shown to not only increase egg production and oviposition rates (Loher & Edson, 1973; Murtaugh & Denlinger, 1985) but also increase disease resistance to bacterial pathogens as well (Shoemaker et al., 2006b; Worthington & Kelly, 2016b). Therefore, even if a trade‐off does exist between fecundity and immune function, females in the wild may be able to mediate it by mating frequently to increase access to fitness‐enhancing substances (Worthington et al., 2015; Worthington & Kelly, 2016a).

Interestingly, although mating grants females an immune advantage and there is little evidence of sickness behavior in field crickets (Kelly & Mc Cabe Leroux, 2020; Sullivan et al., 2016), we found that immune‐challenged adults exhibited decreased rates of mating and consequently lower egg viability compared to healthy females. These findings confirm that illness just prior to a mating opportunity can have significant negative impacts on female fitness beyond those predicted by resource constraints alone. Although broader evidence of sickness behavior has not been observed in field crickets, illness may alter female receptivity to mating while fighting off an infection. Importantly, we found that egg production and oviposition rates were unaffected, but that changes to egg viability were negatively impacted. This finding highlights the importance of incorporating more accurate measures of fitness into experimental design so that any consequences of an immune response do not go undetected. The standard practice of pairing males with females for a specified period of time and assuming they mate may yield inaccurate results if mating is not verified. A successful mating should only be counted if the copulation event was directly observed (Worthington & Kelly, 2016b), the female's spermatheca is investigated for sperm following cohabitation with a male (Miyashita et al., 2019; Worthington & Kelly, 2016a), or fertilized eggs or hatchlings are a direct result of a single mating (Shoemaker & Adamo, 2007). Furthermore, while the number of eggs produced or laid are easy to quantify, they too provide an incomplete picture of an individual's fitness if reduced viability or hatchling success limits the number of offspring that are actually produced. Finally, although the immediate impact of illness on fitness is limited, intergenerational effects such as offspring immune status (McNamara, Van Lieshout, et al., 2014) also needs to be taken into consideration to get a comprehensive view of the impact that illness has on populations.

Surprisingly, being anesthetized and handled had an impact on a number of fitness‐related traits. Both control and experimental individuals in the juvenile treatments had reduced mass gain and smaller adult body size, whereas all individuals in the adult treatments suffered from reduced viability of sperm stored within their spermatheca. Ethanol toxicity from sterilizing the abdomen prior to injection was unlikely the cause of these consequences due to the exposure lasting <10 s and only being applied externally; however, the effect of short‐term ethanol exposure should be investigated for future studies. Additionally, briefly using cold temperatures to anesthetize crickets has no effect on the levels of the stress hormone octopamine (OA) or immune function (Adamo & Parsons, 2006), such that long‐term changes to juvenile growth trajectories due to an acute cold stress is unlikely. Likewise, because adult females were not mated until at least 2 days after treatment, there were no treatment differences in sperm exposure to cold prior to the sperm viability analysis. One possible explanation remains – physical restraint has been shown to increase stress responses in insects (Libersat & Pflueger, 2004; Orchard, 1982) and chronic stress in response to arrhythmic vibration results in lower weight gain in *G*. *texensis* (Adamo & Baker, 2011). While all crickets in our study were photographed at the start of their antepenultimate, penultimate, and adult instars to monitor growth patterns, the added stress of restraint during sterilization and injection procedures may have been an acute stressor at the time of treatment, leading to the negative impacts that we observed. Whether acute stressors can have the same long‐term impacts that we see of chronic stress remains to be seen, however. It is important to note that we have used these anesthetization, sterilization, and handling techniques in previous studies and have never documented any negative impacts of them on cricket behavior, physiology, or variables being quantified (Worthington & Kelly, 2016).

In conclusion, we found that although an immune challenge does have negative impacts on reproduction, adults experience fewer fitness trade‐offs than are generally predicted, and that juveniles experience few, if any, reproductive consequences later in life from an acute immune challenge. Past studies have demonstrated that enduring multiple stressors simultaneously can induce complicated physiological interactions (Adamo, 2020; Adamo & McKee, 2017), and here we show that even minor acute stressors can have significant impacts on an individual's growth and reproduction. Together, these findings highlight that factors affecting fitness can be complex and at times unpredictable, and that we must strive for a multifaceted approach to understanding the constraints and adaptations that organisms experience in response to the numerous immune and physiological stressors encountered throughout their lives.

## CONFLICT OF INTEREST

The authors certify they have no affiliations with any organization or entity with financial or non‐financial interests in the subject matter discussed in this manuscript.

## AUTHOR CONTRIBUTIONS

**Daniel J. Breiner:** Conceptualization (equal); Data curation (equal); Funding acquisition (equal); Investigation (lead); Methodology (equal); Project administration (equal); Writing – original draft (lead); Writing – review & editing (supporting). **Matthew R. Whalen:** Data curation (equal); Formal analysis (lead); Visualization (lead); Writing – review & editing (supporting). **Amy M. Worthington:** Conceptualization (equal); Data curation (equal); Formal analysis (supporting); Funding acquisition (equal); Investigation (supporting); Methodology (equal); Project administration (equal); Resources (lead); Supervision (lead); Visualization (supporting); Writing – original draft (supporting); Writing – review & editing (lead).

## Data Availability

All data and R markdown is archived in the Dryad data repository https://doi.org/10.5061/dryad.xd2547djt.
